# Microvasculopathy Evaluated by Dual-Energy Computed Tomography in Patients with Chronic Thromboembolic Pulmonary Hypertension and Pulmonary Arterial Hypertension

**DOI:** 10.3390/life12081232

**Published:** 2022-08-15

**Authors:** Keisuke Miwa, Yu Taniguchi, Hiroyuki Fujii, Yoichiro Matsuoka, Hiroyuki Onishi, Kenichi Yanaka, Yu Izawa, Yasunori Tsuboi, Atsushi Kono, Noriaki Emoto, Kenichi Hirata

**Affiliations:** 1 Division of Cardiovascular Medicine, Department of Internal Medicine, Kobe University Graduate School of Medicine, Kobe 650-0017, Japan; 2 Department of Radiology, Kobe University Graduate School of Medicine, Kobe 650-0017, Japan

**Keywords:** chronic thromboembolic pulmonary hypertension, pulmonary arterial hypertension, dual-energy computed tomography, poor subpleural perfusion, microvasculopathy, balloon pulmonary angioplasty

## Abstract

**Background:** Poor subpleural perfusion (PSP) on dual-energy computed tomography (DE-CT) suggests microvasculopathy in chronic thromboembolic pulmonary hypertension (CTEPH). However, whether the microvasculopathy findings are equivalent to those in pulmonary arterial hypertension (PAH) remains unclear. The aim of this study was to elucidate the characteristics of microvasculopathy in CTEPH compared to those of that in PAH. **Methods:** We retrospectively reviewed subpleural perfusion on DE-CT and the hemodynamics of 23 patients with PAH and 113 with inoperable CTEPH. Subpleural perfusion on DE-CT was classified as poor (subpleural spaces in all segments with little or no perfusion) or normal. **Results:** PSP was observed in 51% of patients with CTEPH and in 4% of those with PAH (*p* < 0.01). CTEPH patients with PSP had poorer baseline hemodynamics and lower diffusing capacity for carbon monoxide divided by the alveolar volume (DLCO/VA) than those with CTEPH with normal perfusion (pulmonary vascular resistance [PVR]: 768 ± 445 dynes-sec/cm^5^ vs. 463 ± 284 dynes-sec/cm^5^, *p* < 0.01; DLCO/VA, 60.4 ± 16.8% vs. 75.9 ± 15.7%, *p* < 0.001). Despite the existence of PSP, hemodynamics improved to nearly normal in both groups after balloon pulmonary angioplasty. **Conclusions:** PSP on DE-CT, which is one of the specific imaging findings in CTEPH, might suggest a different mechanism of microvasculopathy from that in PAH.

## 1. Introduction

Chronic thromboembolic pulmonary hypertension (CTEPH) is caused by persistent non-resolving, organized thromboemboli in the pulmonary arteries. CTEPH is classified into group 4 of the recent clinical classification of pulmonary hypertension (PH) [1,2]. Recent studies have revealed that apart from the stenosis or obstruction by organized thromboemboli in pulmonary arteries, peripheral microvasculopathy (small pulmonary vessel disease) is also involved in the development of PH in patients with CTEPH [3,4].

The histological changes in microvasculopathy are similar to those observed in idiopathic pulmonary arterial hypertension (PAH), including intimal thickening, intimal fibromuscular proliferation, and intimal fibrosis. In addition, microvasculopathy in CTEPH also consists of diffuse distal thrombosis when bronchial arteries fail to develop [4]. Microvasculopathy is usually suspected when patients with CTEPH have disproportionally poor hemodynamics despite mild perfusion defects [4,5]. The usage of imaging modalities to evaluate microvasculopathy in CTEPH has also been reported, and poor subpleural perfusion (PSP) in the capillary phase of pulmonary digital subtraction angiography is considered to reflect the existence of microvasculopathy, including diffuse distal thrombosis [6,7].

Dual-energy computed tomography (DE-CT) has recently emerged as an imaging modality for evaluating pulmonary artery structure and segmental lung perfusion. DE-CT can produce a sensitive iodine distribution map of the lung fields by using low-and high-tube-voltage X-rays to acquire two different datasets simultaneously [8,9]. The local distribution of the contrast (iodine) represents pulmonary perfusion [10,11]. Moreover, the extent of hypoperfusion in the subpleural area reflected in the color-coded lung perfusion blood volume (lung PBV) images can also be observed by DE-CT. Onishi et al., using a cohort of 93 patients, reported that PSP on DE-CT corresponded with PSP in the capillary phase of pulmonary digital subtraction angiography and that it might also suggest the existence of microvasculopathy in CTEPH [12].

Although the existence of microvasculopathy in CTEPH has been widely suggested, it remains unclear whether its pathological findings are equivalent to those found in PAH. We aimed to elucidate the characteristics of microvasculopathy in CTEPH and compare them to those in PAH by using clinical parameters and DE-CT findings.

## 2. Materials and Methods

This observational study was conducted on patients diagnosed with PAH or non-operable CTEPH who were eligible for balloon pulmonary angioplasty (BPA) and underwent DE-CT at the time of diagnosis at Kobe University Hospital (Kobe, Japan) between July 2013 and April 2020. The diagnoses of PAH or CTEPH were made according to current clinical guidelines during the observational period [13,14]. Right heart catheterization was performed to establish the definitive diagnosis of PH. Additionally, ventilation-perfusion scintigraphy, computed tomography pulmonary angiography with DE-CT, and selective pulmonary angiography were performed to establish the definitive CTEPH diagnosis. The lung function test, arterial blood gas composition, echocardiography, and functional status according to the New York Heart Association functional class (NYHA FC) classification, exercise capacity based on the 6 min walk distance (6MWD), and the cardio-pulmonary exercise test were routinely assessed at the time of diagnosis and 3 months after the last BPA session. Data were collected from hospital medical records.

The primary objective of this study was to clarify the difference between the findings of CTEPH and PAH on DE-CT. The secondary objective was to clarify the clinical impact of PSP in the treatment of CTEPH.

### 2.1. DE-CT Imaging Protocol

DE-CT was performed using a third-generation dual-source CT scanner (SOMATOM Force; Siemens AG, Erlangen, Germany) operating in the dual-energy scan mode, with a tube A voltage of 150 kV, yielding a reference of 208 mA and a tube B voltage of 80 kV, yielding a reference of 374 mA. A 10 mL iodine-containing contrast medium (370 mg I/mL) diluted in half using saline was injected as a test injection to determine the scan delay. The contrast medium was injected for 10 s at a rate of 22 mgI/kg/s. A region of interest (ROI) was mapped out in the main pulmonary artery, and the time-density curve within the ROI was recorded. The early phase DE-CT scan was acquired 4 s after the test injection-mediated enhancement had peaked from the lung’s base to its apex. Composite images were created by fusing the high- and low-voltage images, and color-coded lung PBV images were reconstructed at 5 mm intervals using the dual-energy application software *syngo* CT Workplace, VA44A (Siemens AG, Erlangen, Germany).

### 2.2. Evaluation of Subpleural Perfusion and Lung PBV Score on DE-CT

We assessed the subpleural perfusion on DE-CT in each 5 mm slice using the methodology previously described by Onishi et al. [12]. This methodology is similar to that used for subpleural perfusion assessment in digital subtraction angiography [6,7]. The subpleural area was defined as ≤1.5 cm (approximately the width of one rib) from the lateral pleura in the horizontal section.

**Figure 1:**
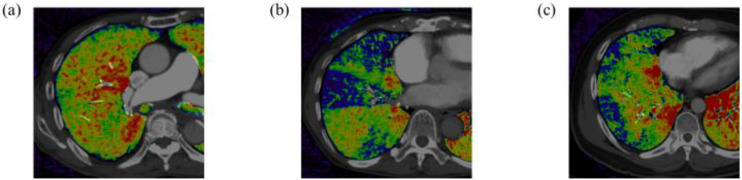
Horizontal section of DE-CT image in a patient with normal subpleural perfusion (**a**), wedge-shaped segmental defect (**b**), and poor subpleural perfusion (**c**). Black-blue color-coded image represents poor lung perfusion, and red-yellow color-coded image represents well lung perfusion.

The subpleural perfusion level on DE-CT was classified into three types: (1) normal perfusion, (2) wedge-shaped segmental defect: no or poor perfusion spread evenly in a wedge shape due to proximal vessel occlusion, and (3) poor perfusion: no or minimal perfusion (Figure 1a–c).

CTEPH patients were assigned to either the poorly perfused or normally perfused groups according to the type of their subpleural perfusion. The normally perfused group included patients with normal perfusion of the subpleural space in at least one segment. The poorly perfused group comprised the patients with subpleural spaces that were non- or minimally perfused in all segments [6,7]. The wedge-shaped segmental defect was considered to be due to proximal vessel occlusion, in which case it was difficult to assess the subpleural perfusion; therefore, the area of a simple wedge-shaped segmental defect was excluded from the subpleural perfusion analysis. In patients with operable CTEPH, the perfusion area was widely defective in a wedge shape since the pulmonary artery was obstructed at either the lobar or the proximal segmental level. In these patients, subpleural perfusion assessment was difficult, and we, therefore, excluded operable CTEPH patients from our study.

Two cardiologists blinded to the patients’ identity performed the subpleural perfusion assessment of DE-CT images. The interobserver agreement was verified by a McNemar test for the first 50 patients (*p* < 0.001). In cases where the assessment was difficult, a final consensus was reached by consulting an experienced cardiologist and a radiologist (YT and AK). Lung perfusion was quantified using the lung PBV score, calculated automatically by the dual-energy application software *syngo* CT Workplace, VA44A (Siemens AG, Erlangen, Germany) as the sum of the iodine density scores of 18 lung segments.

The inclusion criteria were as follows: (a) patients with PAH or non-operable CTEPH who were eligible for BPA; and (b) patients who underwent DE-CT at the time of diagnosis. The exclusion criteria were as follows: (a) patients with PH due to left heart or chronic lung diseases; (b) patients with operable CTEPH, and (c) PAH patients who had previously been treated with PAH-specific drugs or those with CTEPH who had previously undergone pulmonary endarterectomy or BPA.

### 2.3. Statistical Analysis

All statistical analyses were performed using SPSS Statistics (version 26.0; IBM Corp., Armonk, NY, USA). Continuous variables are expressed as mean ± standard deviation or median and interquartile range, according to the variable distribution. Differences in continuous variables, such as patient age, 6 MWD, lung PBV score, hemodynamic characteristics, exercise capacity, and lung function, were compared using the independent Student’s t-test for normally distributed and the Mann–Whitney U test for non-normally distributed variables. Categorical data on sex, NYHA FC classification, and use of PAH-targeted medication are expressed as numbers and percentages and were compared using the χ2 test for independence. For all analyses, the level of statistical significance was set at *p* < 0.05.

## 3. Results

**Figure 2:**
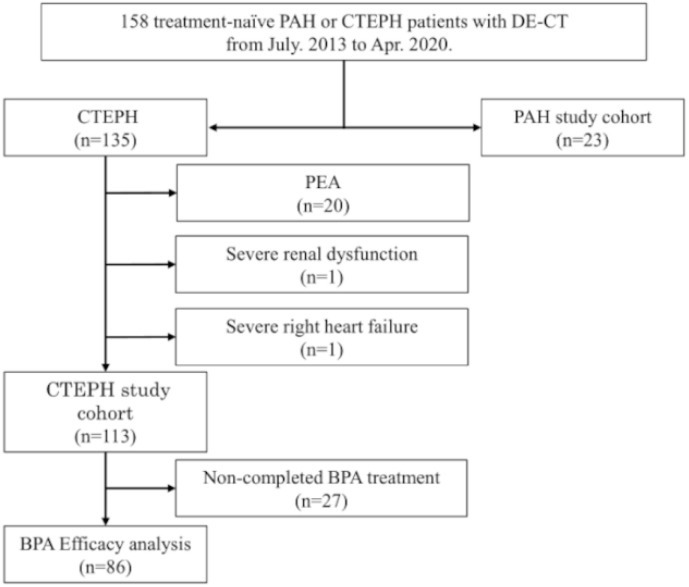
Study flow chart.

During the study period, 135 and 23 patients were diagnosed with CTEPH and PAH, respectively. Among the patients with CTEPH, 20 were considered operable, underwent pulmonary endarterectomy, and were then excluded from the study. Among the remaining 115 patients, two did not undergo DE-CT: one due to renal dysfunction and the other due to severe right heart failure. The remaining 113 patients with CTEPH, who were considered inoperable and underwent BPA, were enrolled. Of them, 86 completed adequate BPA treatment and underwent re-evaluation of hemodynamic assessment by right heart catheterization (RHC) after a median of 85.8 days (IQR: 65.1; 104.2 days) following the last BPA session. The study flow chart is shown in Figure 2.

**Figure 3:**
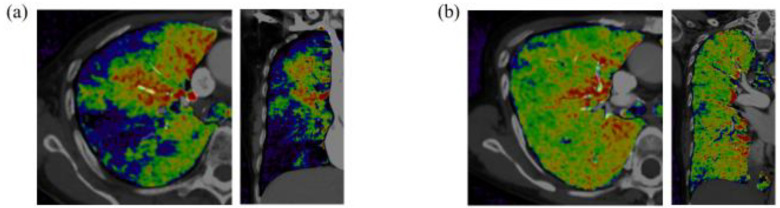
Representative horizontal section and coronal section of DE-CT images in a patient with CTEPH with poor subpleural perfusion (**a**) and PAH (**b**). Black-blue color-coded image represents poor lung perfusion, and red-yellow color-coded image represents well lung perfusion.

The patients’ baseline characteristics and hemodynamics are shown in Table 1. Both were similar between the patients with PAH and those with CTEPH. PSP was observed in more than half of the patients with CTEPH, whereas it was rarely observed in those with PAH (51% vs. 4%, *p* < 0.001). Representative images of DE-CT in a patient with CTEPH and in one with PAH are shown in Figure 3a,b.

### 3.1. Lung PBV Score and Pulmonary Vascular Resistance

**Figure 4:**
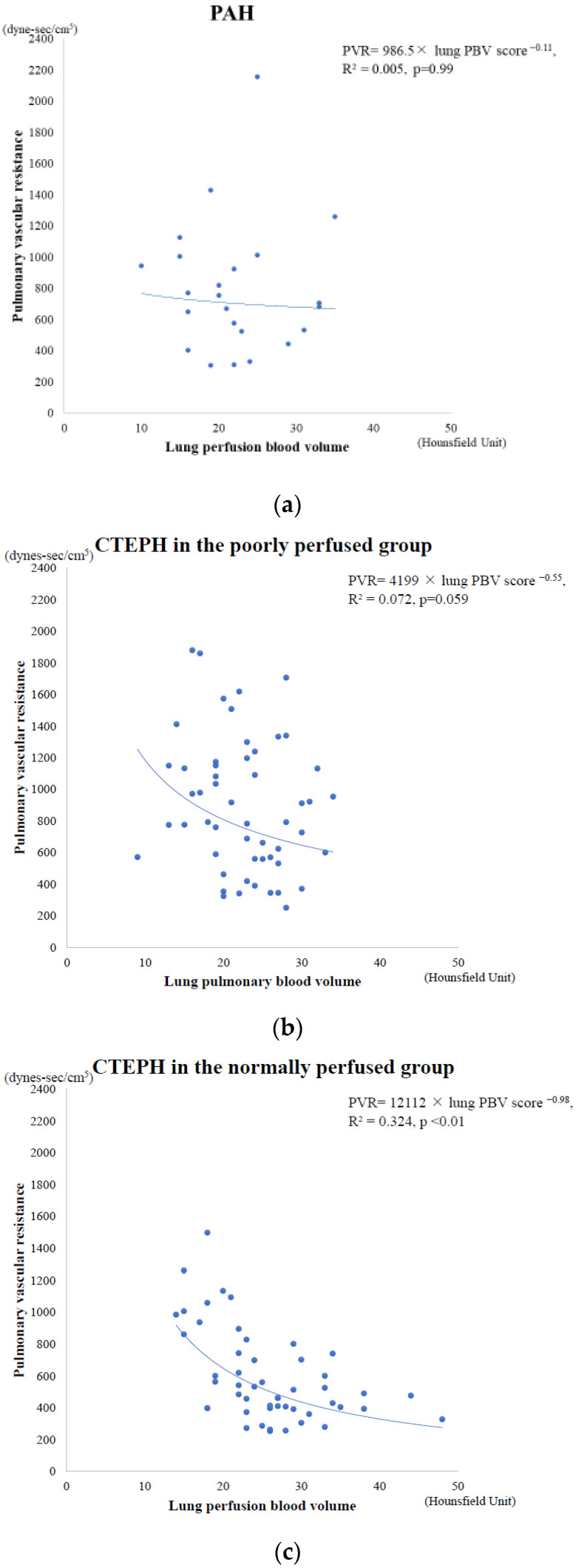
The relationship between PVR obtained through right heart catheterization and lung PBV score obtained using DE-CT in (**a**) patients with PAH (*n* = 23) (PVR = 986.5 × lung PBV score^−0.11^, R^2^ = 0.005, *p* = 0.99), (**b**) CTEPH patients in the poorly perfused group (*n* = 58) (PVR = 4199 × lung PBV score^−0.55^, R^2^ = 0.072, *p* = 0.059), and (**c**) CTEPH patients in the normally perfused group (*n* = 55) (PVR = 12,112 × lung PBV score^−0.98^, R^2^ = 0.324, *p* < 0.01).

Of the 113 patients with CTEPH included in the analysis, 58 (51%) with PSP on DE-CT were assigned to the poorly perfused group, and 55 (49%) without PSP were assigned to the normally perfused group. Figure 4 shows the relationship between the lung PBV score and pulmonary vascular resistance (PVR) of (a) patients with PAH, (b) patients with CTEPH in the poorly perfused group, and (c) patients with CTEPH in the normally perfused group. In the patients with PAH and those with CTEPH in the poorly perfused group, there was no significant correlation between the lung PBV score and PVR (PVR = 986.5 × lung PBV score^−0.11^, R^2^ = 0.005, *p* = 0.99, and PVR = 4199 × lung PBV score^−0.55^, R^2^ = 0.072, *p* = 0.059, respectively); however, a strong nonlinear and inverse correlation between the lung PBV scores and PVR was observed in patients with CTEPH in the normally perfused group (PVR = 12,112 × lung PBV score^−0.98^, R^2^ = 0.324, *p* < 0.01).

### 3.2. Hemodynamic Results of CTEPH According to Subpleural Perfusion on DE-CT

The baseline clinical characteristics and hemodynamics of patients with CTEPH in the normally perfused and poorly perfused groups are summarized in Table 2. Patient characteristics, including age, sex ratio, and functional status, were similar between the groups. Patients in the poorly perfused group had worse hemodynamics with higher PVR (768 ± 445 dynes-sec/cm^5^ vs. 463 ± 284 dynes-sec/cm^5^, *p* < 0.01) and lower SvO_2_ (60.3 ± 8.2% vs. 64.9 ± 7.4%, *p* < 0.01), and they also had a lower diffusing capacity for carbon monoxide divided by alveolar volume (DLCO/VA) (60.4 ± 16.8% vs. 75.9 ± 15.7%, *p* < 0.01). Of the 58 patients, 27 (47%) in the poorly perfused group and 26 of the 55 patients (47%) in the normally perfused group were treated with a soluble guanylate cyclase stimulator (riociguat, an approved drug for CTEPH).

Table 3 summarizes the hemodynamic results of patients in both groups who received adequate BPA and underwent re-evaluation using RHC (*n* = 86). Hemodynamic parameters improved to nearly normal in both groups (PVR: 611 ± 467 dynes-sec/cm^5^ to 270 ± 118 dynes-sec/cm^5^, *p* < 0.001, mean pulmonary arterial pressure (PAP): 37.5 ± 10.5 mmHg to 19.6 ± 5.0 mmHg, *p* < 0.001 in the poorly perfused group; PVR: 422 ± 280 dynes-sec/cm^5^ to 220 ± 88 dynes-sec/cm^5^, *p* < 0.001, mean PAP: 32.5 ± 11.1 mmHg to 19.2 ± 3.3 mmHg, *p* < 0.001 in the normally perfused group). In the poorly perfused group, exercise capacities, i.e., 6 min walk distance, and peak VO_2_ in the cardio-pulmonary exercise test, were also improved. However, the poorly perfused group had a higher VE/VCO_2_ slope (28.8 ± 5.9 vs. 25.9 ± 4.5, *p* = 0.040) and lower %DLCO/VA (55.5 ± 13.1% vs. 70.0 ± 12.4%, *p* = 0.001) than the normally perfused group, even after adequate BPA.

## 4. Discussion

In this study, PSP on DE-CT was observed in more than half of the patients with CTEPH; however, it was rarely observed in patients with PAH. The detection of PSP on DE-CT, a specific imaging finding in CTEPH, might suggest a mechanism of microvasculopathy in CTEPH (microvasculopathy of extensive distal thrombosis) different from that found in PAH. DE-CT might be useful for assessing microvasculopathy of diffuse distal thrombosis with PSP in CTEPH.

Digital subtraction pulmonary angiography, an invasive imaging modality, is considered the gold standard for characterizing vessel morphology in CTEPH.

Current guidelines for CTEPH indicate the usefulness of ventilation/perfusion scintigraphy as a screening tool due to its high sensitivity and specificity of 95–97% and 90–95%, respectively [13,15]. Recent studies have shown the diagnostic accuracy of computed tomography pulmonary angiography and dynamic contrast-enhanced lung perfusion magnetic resonance imaging as noninvasive modalities [16,17,18]. In addition, DE-CT is also emerging as a valuable modality for outlining the pulmonary vasculature. Several reports have already supported the diagnostic accuracy of DE-CT and concur with the usefulness of ventilation/perfusion scintigraphy and computed tomography pulmonary angiography in CTEPH [19,20]. Lung vascular perfusion can be quantified by examining the lung PBV score, which is calculated as the sum of the iodine density scores of each lung segment. Additionally, PBV maps could calculate the iodine distribution in the lung parenchyma and be used as surrogate markers for the underlying vascular reserve [5]. Takagi et al. reported that the lung PBV score could be a noninvasive way of estimating the clinical severity of CTEPH, since it is significantly associated with hemodynamic parameters, including mean PAP and PVR. [11]. Onishi et al. reported the possibility of using DE-CT not only to quantify pulmonary vascular perfusion with the lung PBV score but also to qualitatively evaluate microvasculopathy using a more sensitive analysis of PSP in three dimensions [12]. Therefore, DE-CT might be useful for evaluating CTEPH severity, pulmonary vascular perfusion, and microvasculopathy.

This study was based on the assessment of subpleural perfusion on DE-CT; the existence of microvasculopathy in CTEPH was not verified by histological examination. PSP reflects the diffuse reduction of pulmonary flow in the peripheral micro vessels, which was observed in 58 of the 113 patients in our study. Although a wedge-shaped segmental defect, likely visible due to proximal vessel occlusion, would be the typical DE-CT finding [20], PSP on DE-CT was observed in more than half of the CTEPH patients; therefore, this might be one of the specific imaging findings in CTEPH.

PSP on digital subtraction angiography or DE-CT might suggest the presence of microvasculopathy and/or diffuse distal thrombosis [6,7,12]. Indeed, patients with PSP showed poor hemodynamics, disproportionate to the degree of pulmonary vascular obstruction. Additionally, not only pulmonary vascular obstruction but also microvasculopathy may contribute to severe hemodynamic instability [3,4]. However, PSP is rarely observed in patients with PAH, a disease characterized by obliteration and remodeling of the small pulmonary arteries [2,13]. PSP in CTEPH may not suggest the same kind of microvasculopathy as the one found in PAH. Histopathological examination by biopsy and autopsy in CTEPH patients not only revealed PAH-like lesions (including intimal fibromuscular proliferation or plexiform lesion), but thrombotic lesions in pulmonary arteriole [21], and these lesions were also observed in pulmonary veins and capillaries [22]. Microvasculopathy in CTEPH also comprises diffuse distal thrombosis [4], which might be represented by PSP on DE-CT, as it reflects the diffusely reduced pulmonary flow in peripheral micro vessels.

Simonneau et al. suggested that distal thrombosis in CTEPH could be diffuse when the patency of small pulmonary arterioles distal to complete obstructions are not maintained because bronchial arteries and anastomoses fail to develop [4]. In a report by Taniguchi et al., PSP in digital subtraction angiography was also found to be associated with less developed bronchial arteries, suggesting that bronchial-pulmonary anastomoses have the role of maintaining the patency of the pulmonary capillary bed distal to the obstructed pulmonary artery in CTEPH [7]. In this study, we showed that more than half of the patients with CTEPH also had diffusely reduced pulmonary flow in peripheral micro vessels.

The clinical impact of PSP in CTEPH remains unknown. In this study, the lung PBV score, which could represent lung vascular perfusion of the pulmonary artery, showed a strong inverse correlation with PVR in CTEPH without PSP; however, there was no significant correlation between the lung PBV score and PVR in CTEPH with PSP, nor in PAH. This might suggest that not only pulmonary vascular obstruction by thromboemboli, but also diffuse distal thrombosis is involved in the CTEPH hemodynamics. The data might be comparable to those reported by Azarian et al., which demonstrated no significant correlation between the percentage of vascular obstruction and total pulmonary resistance in patients with CTEPH [23].

However, this study demonstrated that hemodynamics at rest improved significantly after adequate BPA in patients with or without PSP (poorly perfused group: mean PAP 19.6 ± 5.0 mmHg, PVR 270 ± 118 dynes-sec/cm^5^; normally perfused group: mean PAP 19.2 ± 3.3 mmHg, PVR 220 ± 88 dynes-sec/cm^5^). Several reports have found that PAP at rest does not increase unless more than 50% of the pulmonary microcirculation is lost [24]. In patients with CTEPH, pulmonary circulation does not completely return to normal, even after optimal and apparently successful surgical endarterectomy, nor after interventional or medical treatment [22,25]. Kikuchi et al. reported that 47% of CTEPH patients who had completely normalized mean PAP at rest after BPA showed exercise PH. The cause for this might have been a small vessel disease that was not treatable by BPA [26]. The European Respiratory Society Task Force on CTEPH stated that in many patients with CTEPH, resting mean PAP is normalized by surgery or multimodal treatment, and patients felt healthy; however, it is unlikely to return all pulmonary vessels back to normal [5]. VE/VCO2, which is a marker of ventilatory inefficiency and reflects the ventilation-perfusion mismatch [27], remained elevated, and %DLCO/VA, which was associated with poor outcomes in patients with CTEPH and might indicate a pronounced microvasculopathy [7,28], remained low in the PSP group, even though all accessible lesions had been treated after adequate BPA. However, in patients in the poorly perfused group, we considered that hemodynamics at rest improved to nearly normal, despite being poor at baseline. The proximal pulmonary artery flow improved following BPA even though pulmonary microcirculation remained impaired. Therefore, BPA should be considered a first-line treatment for inoperable CTEPH, regardless of the existence of PSP on DE-CT. Further investigations are needed to better understand peripheral microcirculation in CTEPH.

## 5. Limitations

The main limitation of this study was its retrospective observational design. This was a single-center study with a relatively small sample size, which inevitably leads to an increased possibility of selection bias. Moreover, this study was based on the assessment of subpleural perfusion on DE-CT; the existence of microvasculopathy in CTEPH was not verified by histological examination, which is the gold standard, because autopsies and biopsies could not have been performed due to ethical limitations. Another limitation is that we did not assess the efficacy of the sGC stimulator, which might affect small vessel diseases other than diffuse microthrombosis.

## 6. Conclusions

PSP presence on DE-CT, which reflects diffusely reduced pulmonary flow in peripheral micro vessels, might suggest a mechanism of microvasculopathy in CTEPH (microvasculopathy of extensive distal thrombosis) different from that found in PAH. PSP on DE-CT was observed in more than half of the patients with CTEPH; therefore, it might be a specific CTEPH imaging finding. However, hemodynamics at rest improved to nearly normal after BPA treatment despite the existence of PSP reflecting the diffuse reduction in pulmonary flow in the peripheral micro vessels.

## Figures and Tables

**Table 1:** Hemodynamics and characteristics of patients with PAH and CTEPH at diagnosis.

Variable	PAH (*n* = 23)	CTEPH (*n* = 113)	*p* Value
*Baseline characteristics*			
Age (years)	68 ± 14	70 ± 13	0.450
Male (*n*, %)	10 (43)	27 (24)	0.054
NYHA FC (I/II/III/IV) (%)	4/39/53/4	1/20/72/7	0.128
BNP (pg/mL)	170 [210]	263 [204]	0.208
DE-CT parameters			
Poor subpleural perfusion (*n*, %)	1 (4%)	58 (51%)	<0.001
Lung PBV score (Hounsfield Unit)	21.5 ± 6.51	24.0 ± 6.5	0.188
*Baseline hemodynamics*			
Mean RAP (mmHg)	4.0 ± 3.3	5.0 ± 3.6	0.963
Systolic PAP (mmHg)	59 ± 13.2	63 ± 18.7	0.071
Diastolic PAP (mmHg)	25 ± 7.5	22 ± 7.8	0.138
Mean PAP (mmHg)	38 ± 8.3	37 ± 10.9	0.826
PAWP (mmHg)	8.0 ± 3.8	8.0 ± 3.8	0.826
Cardiac output (L/min)	3.16 ± 0.76	3.54 ± 2.22	<0.001
Cardiac index (L/min/m^2^)	2.12 ± 0.43	2.06 ± 0.78	0.216
PVR (dynes-sec/cm^5^)	703 ± 413	564 ± 395	0.207
*Exercise capacity*			
6MWD (m)	300 ± 116	318 ± 97	0.416
Peak VO_2_ in CPET (mL/min/kg)	13 ± 5.2	12.4 ± 4.1	0.526
VE/VCO_2_ slope in CPET	37.3 ± 14.2	39.1 ± 11.5	0.697
*Lung function test*			
%VC (%)	89.6 ± 20.0	90.35 ± 17.3	0.691
FEV 1.0% (%)	84.9 ± 17.3	85.4 ± 18.7	0.325
%DLCO/VA (%)	39.8 ± 22.0	70.1 ± 17.5	<0.001

*List of abbreviations*: NYHA FC: New York Heart Association functional class; BNP: brain natriuretic peptide; PBV: pulmonary blood volume; RAP: right atrial pressure; PAP: pulmonary artery pressure; PAWP: pulmonary artery wedge pressure; PVR: pulmonary vascular resistance; 6MWD: 6 min walk distance; VO_2_: oxygen consumption; CPET: cardio-pulmonary exercise test; VE/VCO_2_: ventilatory equivalent for carbon dioxide; VC: vital capacity; FEV: forced vital capacity; DL_CO_/VA: diffusing capacity for carbon monoxide divided by the alveolar volume. Data are given as mean ± standard deviation or median [interquartile range].

**Table 2:** Baseline characteristics and treatments in patients with CTEPH in the normally and poorly perfused groups.

Variable	Poorly Perfused *n* = 58	Normally Perfused *n* = 55	*p* Value
*Baseline characteristics*			
Age (years)	70 ± 12	71 ± 13	0.700
Male (*n*, %)	15 (26%)	12 (22%)	0.663
NYHA FC (I/II/III/IV) (%)	0/17/72/10	2/24/71/4	0.334
*Baseline hemodynamics*			
Mean RAP (mmHg)	4.0 ± 3.9	5.0 ± 3.3	0.835
Systolic PAP (mmHg)	70.0 ± 16.8	60.0 ± 19.6	0.018
Diastolic PAP (mmHg)	25.2 ± 7.7	20.0 ± 7.7	0.032
Mean PAP (mmHg)	39.2 ± 10.2	34.1 ± 11.1	0.052
PAWP (mmHg)	8.3 ± 3.5	8.0 ± 4.1	0.191
Cardiac output (L/min)	3.86 ± 1.93	3.20 ± 2.37	0.508
Cardiac index (L/min/m^2^)	1.89 ± 0.78	2.27 ± 0.73	0.023
PVR (dynes-sec/cm^5^)	768 ± 445	463 ± 284	<0.001
SvO_2_ (%)	60.3 ± 8.2	64.9 ± 7.4	0.003
*Exercise capacity*			
6MWD (m)	305 ± 97	355 ± 94	0.097
Peak VO_2_ in CPET (mL/min/kg)	13.5 ± 4.3	11.6 ± 3.9	0.700
VE/VCO_2_ slope in CPET	39.7 ± 11.6	37.1 ± 10.7	0.039
*Lung function test*			
%VC (%)	91.7 ± 16.7	88.3 ± 17.8	0.646
FEV 1.0% (%)	73.1 ± 8.9	76.5 ± 8.7	0.188
%DLCO/VA (%)	60.4 ± 16.8	75.9 ± 15.7	<0.001
*Medications initiated since diagnosis*			
*anticoagulation*			
Warfarin (*n*, %)	40 (69%)	33 (60%)	0.319
DOAC (*n*, %)	18 (31%)	22 (40%)	0.319
*PAH-specific drugs*			
ERA (*n*, %)	9 (16%)	1 (2%)	0.017
PDE5-i (*n*, %)	6 (10%)	0 (0%)	0.027
sGC stimulator (*n*, %)	27 (47%)	26 (47%)	0.939

*List of abbreviations*: NYHA FC: New York Heart Association functional class; BNP: brain natriuretic peptide; PBV: pulmonary blood volume; RAP: right atrial pressure; PAP: pulmonary artery pressure; PAWP: pulmonary artery wedge pressure; PVR: pulmonary vascular resistance; 6MWD: 6 min walk distance; VO_2_: oxygen consumption; CPET: cardio-pulmonary exercise test; VE/VCO_2_: ventilatory equivalent for carbon dioxide; VC: vital capacity; FEV: forced vital capacity; DL_CO_/VA: diffusing capacity for carbon monoxide divided by the alveolar volume; DOAC: direct oral anticoagulants; ERA: endothelin-receptor antagonists; PDE5-i: phosphodiesterase type-5 inhibitors; sGC: soluble guanylate cyclase; Data are given as mean ± standard deviation.

**Table 3:** Hemodynamic results of CTEPH patients with BPA (*n* = 86) between the poorly and normally perfused groups.

	Poorly Perfused Group (*n* = 38)			Normally Perfused Group (*n* = 48)			
Variable	Baseline	After BPA	*p* Value	Baseline	After BPA	*p* Value	*p* Value *
Number of BPA session		4.1 ± 2.2			4.4 ± 1.6		0.235
NYHA FC (I/II/III/IV) (%)	0/17/73/10	34/58/8/0	<0.001	2/23/71/4	42/56/2/0	<0.001	0.395
Hemodynamics after BPA							
Mean RAP (mmHg)	4.4 ± 3.7	3.7 ± 3.5	0.033	5.2 ± 3.3	3.8 ± 2.4	0.030	0.712
Systolic PAP (mmHg)	68.5 ± 16.9	34.5 ± 7.7	<0.001	57.0 ± 19.3	32.3 ± 6.5	<0.001	0.090
Diastolic PAP (mmHg)	22.0 ± 7.3	11.3 ± 3.5	<0.001	19.5 ± 7.3	11.1 ± 3.5	<0.001	0.766
Mean PAP (mmHg)	37.5 ± 10.5	19.6 ± 5.0	<0.001	32.5 ± 11.1	19.2 ± 3.3	<0.001	0.220
PAWP (mmHg)	7.9 ± 3.5	7.5 ± 3.5	0.387	8.2 ± 4.1	8.1 ± 3.4	0.433	0.650
Cardiac index (L/min/m^2^)	1.95 ± 0.85	2.48 ± 0.64	0.013	2.32 ± 0.74	2.29 ± 0.71	0.398	0.419
PVR (dynes-sec/cm^5^)	611 ± 467	270 ± 118	<0.001	422 ± 280	220 ± 88	<0.001	0.133
SvO_2_ (%)	61.0 ± 7.7	67.8 ± 4.4	<0.001	65.1 ± 7.4	68.7 ± 5.3	0.013	0.800
Exercise capacity after BPA							
6MWD (m)	300 ± 97	390 ± 102	<0.001	352 ± 93	365 ± 69	0.185	0.627
Peak VO_2_ in CPET (ml/min/kg)	11.1 ± 3.8	15.0 ± 4.5	<0.001	13.4 ± 4.4	15.4 ± 3.6	0.005	0.888
VE/VCO_2_ slope in CPET	39.4 ± 12.7	28.8 ± 5.9	<0.001	35.3 ± 11.1	25.9 ± 4.5	<0.001	0.040
Lung function test							
%VC (%)	91.7 ± 16.7	100.8 ± 20.7	0.065	88.3 ± 17.8	94.2 ± 14.8	0.190	0.821
FEV 1.0% (%)	73.1 ± 8.9	77.8 ± 8.5	0.164	76.5 ± 8.7	76.7 ± 9.2	0.702	0.629
%DLCO/VA (%)	60.4 ± 16.8	55.5 ± 13.1	0.141	75.9 ± 15.7	70.0 ± 12.4	0.040	0.001
Medication							
sGC stimulator (*n*, %)	19 (50%)	18 (47%)		23 (48%)	21 (44%)		0.738

*List of abbreviations*: NYHA FC: New York Heart Association functional class; RAP: right atrial pressure; PAP: pulmonary artery pressure; PAWP: pulmonary artery wedge pressure; PVR: pulmonary vascular resistance; SvO_2_: mixed venous oxygen saturation; 6MWD: 6 min walk distance; VO_2_: oxygen consumption; CPET: cardio-pulmonary exercise test; VE/VCO_2_: ventilatory equivalent for carbon dioxide; VC: vital capacity; FEV: forced vital capacity; DL_CO_/VA: diffusing capacity for carbon monoxide divided by the alveolar volume; sGC: soluble guanylate cyclase; Data are given as mean ± standard deviation. * Comparison between poorly perfused group and normally perfused group after BPA.

## Data Availability

The data presented in this study are available on request from the corresponding author. The data are not publicly available due to privacy.
